# Anti‐atherosclerotic effect of the angiotensin 1–7 mimetic AVE0991 is mediated by inhibition of perivascular and plaque inflammation in early atherosclerosis

**DOI:** 10.1111/bph.13685

**Published:** 2017-02-01

**Authors:** D S Skiba, R Nosalski, T P Mikolajczyk, M Siedlinski, F J Rios, A C Montezano, J Jawien, R Olszanecki, R Korbut, M Czesnikiewicz‐Guzik, R M Touyz, T J Guzik

**Affiliations:** ^1^ Department of Internal and Agricultural Medicine Jagiellonian University School of Medicine Krakow Poland; ^2^ Institute of Cardiovascular and Medical Sciences University of Glasgow UK; ^3^ Department of Pharmacology Jagiellonian University School of Medicine Krakow Poland

## Abstract

**Background and Purpose:**

Inflammation plays a key role in atherosclerosis. The protective role of angiotensin 1–7 (Ang‐(1–7)) in vascular pathologies suggested the therapeutic use of low MW, non‐peptide Ang‐(1–7) mimetics, such as AVE0991. The mechanisms underlying the vaso‐protective effects of AVE0991, a Mas receptor agonist, remain to be explored.

**Experimental Approach:**

We investigated the effects of AVE0991 on the spontaneous atherosclerosis in apolipoprotein E (ApoE)−/− mice, in the context of vascular inflammation and plaque stability.

**Key Results:**

AVE0991 has significant anti‐atherosclerotic properties in ApoE−/− mice and increases plaque stability, by reducing plaque macrophage content, without effects on collagen. Using the descending aorta of chow‐fed ApoE−/− mice, before significant atherosclerotic plaque develops, we gained insight to early events in atherosclerosis. Interestingly, perivascular adipose tissue (PVAT) and adventitial infiltration with macrophages and T‐cells precedes atherosclerotic plaque or the impairment of endothelium‐dependent NO bioavailability (a measure of endothelial function). AVE0991 inhibited perivascular inflammation, by reducing chemokine expression in PVAT and through direct actions on monocytes/macrophages inhibiting their activation, characterized by production of IL‐1β, TNF‐α, CCL2 and CXCL10, and differentiation to M1 phenotype. Pretreatment with AVE0991 inhibited migration of THP‐1 monocytes towards supernatants of activated adipocytes (SW872). Mas receptors were expressed in PVAT and in THP‐1 cells *in vitro*, and the anti‐inflammatory effects of AVE0991 were partly Mas dependent.

**Conclusions and Implications:**

The selective Mas receptor agonist AVE0991 exhibited anti‐atherosclerotic and anti‐inflammatory actions, affecting monocyte/macrophage differentiation and recruitment to the perivascular space during early stages of atherosclerosis in ApoE−/− mice.

**Linked Articles:**

This article is part of a themed section on Targeting Inflammation to Reduce Cardiovascular Disease Risk. To view the other articles in this section visit http://onlinelibrary.wiley.com/doi/10.1111/bph.v174.22/issuetoc and http://onlinelibrary.wiley.com/doi/10.1111/bcp.v82.4/issuetoc

**Abbreviations**
Ang‐(1–7): angiotensin 1–7
Ang II: angiotensin II
ApoE: apolipoprotein E
AT: adipose tissue
ICAM‐1: intercellular adhesion molecule 1
NK: natural killer
PVAT: perivascular adipose tissue
α‐SMA: α‐smooth muscle actin
SNP: sodium nitroprusside

## Tables of Links

**Table:** 

**TARGETS**
**Other protein targets** ^*a*^
TNF‐α
**GPCRs** ^*b*^
CCR7
CXCR3
Mas1 receptor

**LIGANDS**	
AVE0991	IFN‐γ
A779, [D‐Ala7]‐angiotensin(1‐7)	IL‐1β
Ang‐(1‐7), angiotensin‐(1‐7)	IL‐6
CCL2	IL‐8
CCL5	Indomethacin
CXCL10	Insulin
Dexamethasone	LPS
IBMX	PGF_2α_
ICAM‐1	

These Tables list key protein targets and ligands in this article that are hyperlinked to corresponding entries in http://www.guidetopharmacology.org, the common portal for data from the IUPHAR/BPS Guide to PHARMACOLOGY (Southan *et al.,* 2016), and are permanently archived in the Concise Guide to PHARMACOLOGY 2015/16 (^*a,b*^Alexander *et al*., 2015a,b).

## Introduction

Immune activation and inflammation are known to modulate the development of atherosclerotic plaques (Libby *et al.,* 2011; Weber and Noels, 2011; Back and Hansson, 2015) and to regulate plaque stability (Chen *et al.,* 2013). Recent studies suggest that, in very advanced atherosclerotic plaques, tertiary lymphoid structures are formed in the adventitia and in perivascular tissues and may play modulatory, potentially protective role (Hu *et al.,* 2015). In contrast, the importance of perivascular immune cell infiltration at earlier stages of the development of atherosclerotic plaques may suggest a pro‐atherosclerotic role (Galkina *et al.,* 2006; Clement *et al.,* 2015), although immune and inflammatory events at very early stages of the disease, during the process of initiation and maintenance of plaque formation remain poorly defined.

Although much experimental evidence supports the role of the immune system of atherosclerosis, this has not been adequately translated into therapeutic strategies in humans (Libby *et al.,* 2011; Weber and Noels, 2011; Back and Hansson, 2015). This is mainly caused by the view that systemic immunosuppression would be unacceptable for prevention and treatment of cardiovascular diseases. A limited number of clinical approaches are now being studied, including systemic methotrexate or more recently, anti‐cytokine (anti‐IL‐1β or anti‐TNF‐α) treatments (Ridker and Luscher, 2014). These are based on evidence of high concentrations of these cytokines in advanced plaques and their role as checkpoints in atherosclerosis formation *in vivo* and *in vitro*. Surprisingly, while seminal studies have characterized vascular inflammation in fully formed atherosclerotic plaques (Galkina *et al.,* 2006; Macritchie *et al.,* 2012; Clement *et al.,* 2015; Hu *et al.,* 2015), development of vascular inflammation at early stages of atherosclerosis is less clear. Increased oxidative stress and endothelial vascular dysfunction are thought to be key events preceding atherosclerosis development in humans (Guzik *et al.,* 2000; Heitzer *et al.,* 2001) and in animal models (Laursen *et al.,* 2001). In the present study, we aimed to characterize immune cell infiltration in the vascular wall and perivascular tissue at early stages of spontaneous atherosclerosis development in apolipoprotein E (ApoE)−/− mice, and focused on possibilities for pharmacological modulation of this process.

We also tested the possibility that pharmacological inhibition of such early inflammation would translate into protection from atherosclerosis and into increased plaque stability. We focused on a strategy concomitantly known to modulate inflammation and vascular responses, such as activation of angiotensin 1–7 (Ang‐(1–7)) receptors, as this could provide a viable alternative for anti‐inflammatory interventions in atherosclerosis, as well as other vascular diseases, without concerns associated with significant systemic immunosuppression (Passos‐Silva *et al.,* 2013; Magalhaes *et al.,* 2015).

The peptide Ang‐(1–7) exerts numerous protective effects in the vascular system, including vasodilatory (Ren *et al.,* 2002), antioxidant (Raffai *et al.,* 2011) and anti‐inflammatory effects (Lee *et al.,* 2015). In endothelial cells, it stimulated endothelial NOS (eNOS) and NO production (Sampaio *et al.,* 2006), as well as inhibiting vascular NADPH oxidase expression and activity (Benter *et al.,* 2008). Thus, a combination of such vasoprotective and anti‐inflammatory properties can make Ang‐(1–7) and its mimetics particularly beneficial in immune‐mediated vascular disease, such as atherosclerosis. Indeed, 4 week infusion of Ang‐(1–7) peptide, inhibited development of atherosclerosis and protected against endothelial dysfunction in ApoE−/− mice (Tesanovic *et al.,* 2010). The majority of the protective, vascular and immune effects of Ang‐(1–7) are mediated through activation of the Mas receptor, and this action has been well characterized in numerous tissues including brain, lung, vasculature and liver (Sampaio *et al.,* 2006). Ang‐(1–7) Mas receptors are present on immune cells and modulate decreased cytokine production and activation of macrophages, T‐cells and dendritic cells (Capettini *et al.,* 2012; Oliveira‐Lima *et al.,* 2015). Currently, there is only one selective Mas receptor agonist, AVE0991, which lacks significant affinity for angiotensin AT_2_ receptors and is a non‐peptide compound (Bosnyak *et al.,* 2011). Our preliminary studies have demonstrated anti‐atherosclerotic properties of AVE0991 through agonist activity at Mas receptors (Jawien *et al.,* 2012a,b). These effects are independent of alterations in cholesterol metabolism, as AVE0991 does not lead to any alteration of lipid profile (Jawien *et al.,* 2012a). The mechanisms of the potential anti‐atherosclerotic effects of AVE0991 are not known.

Accordingly, in the present study, we have assessed the efficacy of the selective Mas receptor agonist, AVE0991, to provide protection from atherosclerosis through preventing early vascular and perivascular inflammation. To address mechanisms, we used *in vivo* studies of ApoE−/− mice, fed a normal chow, as well as *in vitro* cultures of vascular and inflammatory cells. We demonstrated that AVE0991 had significant anti‐atherosclerotic properties in ApoE−/− mice, and reduced plaque macrophage content. Using the descending aorta of chow‐fed ApoE−/− mice, before significant atherosclerotic plaque develops, we gained insight into the early events in atherosclerosis. Interestingly, perivascular inflammation and infiltration of macrophages and T‐cells preceded atherosclerotic plaque development and the impairment of endothelium dependent, NO‐mediated vasodilatations. AVE0991 inhibited vascular inflammation, through reduction of chemokine expression in perivascular adipose tissue (PVAT), and through direct actions on monocytes/macrophages inhibiting their activation and polarization towards the M1 phenotype. Thus, through *in vivo* and *in vitro* studies, we demonstrated that the Ang‐(1–7) mimetic AVE0991 exhibited anti‐inflammatory properties affecting monocyte/macrophage differentiation and recruitment to perivascular space at early stages of atherosclerosis in ApoE−/− mice.

## Methods

### Animals and treatment

All animal care and experimental procedures were approved by the Jagiellonian University Ethical Committee on Animal Experiments (no. 102/2012). Animal studies are reported in compliance with the ARRIVE guidelines (Kilkenny *et al*., 2010; McGrath & Lilley, 2015).Seventy eight female C57BL/6JBomTac mice and 104 female ApoE^‐/‐^ mice (B6.129P2‐Apoe<tm1Unc> N11) on the C57BL/6J background were obtained from Taconic (Ejby, Denmark). Mice were maintained on 12 h dark/12 h light cycles in air conditioned rooms (22.5 ± 0.5°C, 50 ± 5% humidity) and access to diet and water *ad libitum*. At the age of 12 weeks, mice were randomly assigned into four groups receiving chow diets, pre‐mixed with placebo or with AVE0991, to deliver a dose of 0.58 μmol·kg^−1^ per day, for the following 1–3 months (Supporting Information Figure 1A). All diets were prepared by Ssniff (Soest, Germany). Group one consisted of C57BL/6J mice fed chow/placebo diet containing placebo, Group two – received chow/AVE0991. Group three consisted of ApoE−/− mice receiving chow/placebo diet while group four were the ApoE−/− mice fed chow/AVE0991. Food intake was closely monitored and did not differ between groups. After killing with CO_2_ inhalation, mice were perfused through the vasculature with saline, and the thoracic and abdominal aorta was carefully dissected.

### Analysis of leukocytes in adipose tissue (AT) and peripheral blood

The PVAT from the thoracic and abdominal aorta was isolated as described by Mikolajczyk *et al.,* (2016). Epididymal fat pads were used as representative of visceral fat. ATs were digested using collagenase type XI (125 U·cm^−3^), collagenase type IS (450 U·cm^−3^) and hyaluronidase IV‐S (60 U·cm^−3^) in PBS with calcium and magnesium for 20 min at 37°C, with regular agitation and mashed through a 70 μm strainer (BD Biosciences, USA) to yield single‐cell suspensions (Mikolajczyk *et al.,* 2016). Cells were stained in FACS buffer for 20 min at 4°C in the dark with the monoclonal antibodies shown in Table S3 (Supporting Information Table S3). Viability staining using BD HorizonTM Fixable Viability Stain 510 (FVS510) showed 90 *±* 3% cell viability following isolation from PVAT and the staining procedure. For analysis of peripheral blood monocytes, 1 mL of blood was obtained by cardiac puncture and total peripheral blood mononuclear cells were isolated by density gradient as described (Mikolajczyk *et al.,* 2016). Cells were analysed by a FACSVerse flow cytometer (BD Biosciences, USA), and data were analysed using Flow Jo v.10 (Ashland, OR, USA). For each experiment, fluorescence‐minus‐one controls (FMO) were performed. In selected experiments, FMO gating strategies were confirmed by isotype controls. The gating strategy is described in Supporting Information Figure 1B.

### Vascular reactivity measurements

Aortic segments from the thoracic part of the descending aorta, 2 mm above the diaphragm, were mounted in organ baths (Multi Wire Myograph System – 620 M; DMT – Denmark), containing Krebs–Henseleit solution (composition: NaCl 118 mM, KCl 4.7 mM, KH_2_PO_4_ 1.2 mM, MgSO_4_ 1.2 mM, CaCl_2_ 2.5 mM, NaHCO_3_ 25 mM, glucose 11.7 mM) and aerated with carbogen (95% O_2_/5% CO_2_) to reach pH 7.4, at 37°C and isometric tension studies were performed as described before (Guzik *et al.,* 2000, 2007a; Vinh *et al.,* 2010; Mikolajczyk *et al.,* 2016). Arterial segments were stretched to 1.5 g and pre‐contracted with PGF_2α_ (average concentration 3μM) to obtain 70% of maximal KCl contraction). Concentration–response relaxation curves to ACh (10^−9^ to 10^−6^ M) were obtained. After washout and 20 min equilibration period, rings were contracted with PGF_2α_ (using the same concentrations as above) and concentration–response curves to sodium nitroprusside (SNP: 10^−9^ to 10^−5^ M) were determined.

### Measurement of mRNA expression

Total RNA was obtained from cells using RNeasy Mini Kit (Qiagen). RNA from AT was obtained using RNeasy Lipid Tissue Mini Kit (Qiagen). Total RNA was measured by Nanodrop 2000 (Thermo Fisher Scientific). Reverse transcription of 1 μg RNA was performed using High Capacity cDNA Reverse Transcription Kit (Applied Biosystems). mRNA expression of chosen genes in PVAT were analysed using TaqMan® probes (Thermo Fisher Scientific) (Supporting Information Table S1) and TaqMan® Real‐Time PCR Master Mix (Thermo Fisher Scientific) Reactions were prepared and run on 384‐well plates on the QuantStudio™ 7 Flex Real‐Time PCR System or Applied Biosystems® 7500 Real‐Time PCR with standard protocol. Expressions of mRNA for some genes were analysed using Fast SYBR® Green Master Mix (Thermo Fisher Scientific) and primers from a table (Eurofins) (Supporting Information Table S2). Calculations were made using QuantStudio™ Real‐Time PCR Software or SDS Software 2.4 Data were normalized to levels of GAPDH mRNA for SYBR® Green and S18 for TaqMan®, and relative quantification was calculated as 2^−ΔΔCt^.

### Atherosclerotic plaque quantification

The heart and ascending aorta were embedded in Tissue‐Tek® O.C.T. Compound (Sakura® Finetek, Japan) and snap‐frozen. Sections (10 μm thick) were collected at 100 μm intervals starting from the appearance of the aortic valves. Sections were air dried and fixed in 4% paraformaldehyde followed by staining with Meyer's haematoxylin and Oil Red O (Sigma‐Aldrich, USA). Stained sections were examined under Olympus BX50 microscope (Olympus, Tokyo, Japan). For each animal, total area of the lesion was measured as a mean from four sections. To evaluate atherosclerotic plaque in whole aorta, *en‐face* technique was used. Aortas were fixed in 4% formaldehyde, opened longitudinally, pinned onto black wax plates and stained with Oil Red O (Sigma‐Aldrich, St. Louis, MO, USA). Images of the aorta were recorded using a Nikon D3100 digital camera. Aortic lesion area and total aortic area were calculated using ImageJ software as described (Jawien *et al.,* 2012a). Three sections from each mouse were reviewed and scored without knowledge of the treatments (blinding).

### Immunohistochemical analysis

Aortic sinus sections were used for CD68 and α‐smooth muscle actin (α‐SMA) detection by immunofluorescence (Jawien *et al.,* 2012a). Sections were air dried and fixed in acetone, rehydrated with PBS before incubation in serum‐free Protein Block (DakoCytomation, Milan, Italy). Sections were stained with rat anti‐mouse anti‐CD68 antibody (1:50; AbSerotec, Oxford, UK) diluted in 1% blocking reagent (Perkin Elmer, Cambridge, UK) and 0.3% Triton X‐100 in PBS overnight before being washed in TNT wash buffer (Tris‐HCl; pH 7.5; 0.15 M NaCl; and 0.05% Tween 20, Sigma‐Aldrich). Monoclonal anti–α‐SMA FITC conjugated (1:100, clone 1A4, Sigma‐Aldrich) was added for 1 h before washing. Sections were incubated with 1:200 Texas Red‐donkey anti‐rat IgG (Jackson ImmunoResearch Laboratories, Soham, UK) for 30 min. VECTASHIELD HardSet Antifade Mounting Medium (Vector Laboratories) was used to stain nuclei. Sections incubated without primary antibody were used as a negative control. Images were recorded using fluorescence microscope (Axio Observer Z.1, Zeiss, Germany) with the ZEN software (Zeiss, Germany). Analyses were carried out using Image J software. Three sections from each mouse were reviewed and scored without knowledge of the treatments (blinding).

### Picrosirius red staining

To quantify collagen within the lesions of the aortic sinus, serial cross sections (8 μm) were cut through the aorta beginning at the origin of the aortic valve leaflets and stained with Picrosirius Red. Serial formalin‐fixed sections were incubated for 2 h in a freshly prepared 0.1% solution of Sirius Red F3B (Sigma‐Aldrich) in saturated aqueous picric acid. After rinsing twice in 0.01 M HCl and distilled water, sections were dehydrated and mounted in Permount (Vector Laboratories). Picrosirius Red staining was analysed by polarization microscopy and calculated using Image J without knowledge of the treatments (blinding).

### THP‐1 cell culture and macrophage differentiation

THP‐1 monocytes (American Type Culture Collection, Manassas, VA) were grown at 37°C in a 5% CO_2_ atmosphere in RPMI 1640 supplemented with 10% FBS, 50 U·cm^−3^ penicillin, and 50 U·cm^−3^ streptomycin (pen/strep). Cells were starved in 1% FBS RPMI 1640 (pen/strep) medium overnight. AVE0991 (1 μM) was added 18 h before stimulation. The Mas receptor antagonist A799 (5 μM) was added 30 min before AVE0991. TNF‐α (10 ng cm^−3^) was added for a 6 h incubation. To obtain differentiation into macrophages, THP‐1 cells were treated with 100 nM PMA for 48 h at 37°C, and medium was replaced with RPMI 1640 10% FCS for another 24 h.

### THP‐1 macrophage polarization and effect of AVE0991

After overnight starving (1% FBS RPMI 1640, pen/strep) resting macrophages (M0) were cultured for 24 h in the presence of 20 ng·cm^−3^ IFN‐γ (PeproTech EC, UK) and 10 ng·cm^−3^ LPS to obtain M1 macrophages. M0 THP‐1 macrophages were parallel stimulated with AVE0991 (1 μM) 1 h prior adding IFN‐γ and LPS for 24 h. The phenotype of macrophages were confirmed by gene expression of markers such as CCR7, and CXCL10 for M1, whereas CD206 and CD209 were used for M2.

### SW872 cell culture and adipocyte differentiation

SW872 cells were grown at 37°C in a 5% CO_2_ atmosphere in DMEM supplemented with 10% FBS, 50 U·mL^−1^ penicillin, and 50 U·mL^−1^ streptomycin. For SW872 differentiation to adipocytes, medium was replaced with 10% FBS DMEM with insulin (1 μM), dexamethasone (0.25 μM) and 3‐isobutyl 1‐methylxanthine (IBMX; 0.5 mM) for 48 h. Medium was replaced with 10% FBS DMEM with insulin (1 μM) for another 48 h. For the next 4 days, medium was replaced with 10% FBS DMEM. A day before stimulation, cells were starved in 1% FBS DMEM. For stimulation, AVE0991 (1 μM) was added 18 h before stimulation with TNF‐α (10 ng·mL^−1^) for 24 h. Supernatants were collected after 1000 x *g* centrifugation for 5 min and stored at −80°C until chemotaxis assay.

### Chemotaxis assay

For TNF‐α stimulations, THP‐1 cells were starved in 1%FBS RPMI 1640 medium (with pen/strep) overnight. AVE0991 were added to THP‐1 cell culture 18 h before TNF‐α (10 ng·cm^−3^), 6 h stimulation. THP‐1 cells were centrifuged for 5 min at 400 x *g* and suspended in DMEM 1%FBS with pen/strep and 1 × 10^6^ cells were added to 8 μm pore Transwell® 6 well Inserts (Corning®, US). Supernatants were added to the bottom part and left for 4 h at 37°C in a 5% CO_2_. Cells from bottom part were collected and counted by flow cytometry (LSRII, BD). Fold change of chemotactic properties were calculated comparing cell number passing through the pores to the THP‐1 cells stimulated with TNF‐α and passing through to supernatant from SW872 cells, without stimulation.

### Data and statistical analysis

The data and statistical analysis in this study comply with the recommendations on experimental design and analysis in pharmacology (Curtis *et al.,* 2015). For comparison of three or more independent groups, one‐way anova was used with a Student–Newman–Keuls *post hoc* test. For comparison of two groups, unpaired two‐tailed *t*‐tests were used. For comparison of the effects of AVE0991 on parameters in different groups of mice, we employed two‐way anova with a Bonferroni *post hoc* test. For comparisons of vascular function in organ chamber experiments, repeated measures anova was used. *P* values <0.05 were considered significant.

### Materials

AVE0991 was a kind gift from Sanofi‐Aventis (Frankfurt, Germany); A799 was from Bachem (Bubendorf, Switzerland); IFN‐γ and TNF‐α were supplied by PeproTech EC (London, UK); insulin was from Cell Applications (San Diego, CA) and PGF_2α_ was supplied by Cayman Chemical Company (Ann Arbor, MI). Sigma‐Aldrich (St Louis, MO) supplied ACh, LPS (from Escherichia coli 0111:B4;), dexamethasone, IBMX and SNP.

## Results

### AVE0991 reduces macrophage infiltration and atherosclerotic plaque in ApoE−/− mice

**Figure 1:**
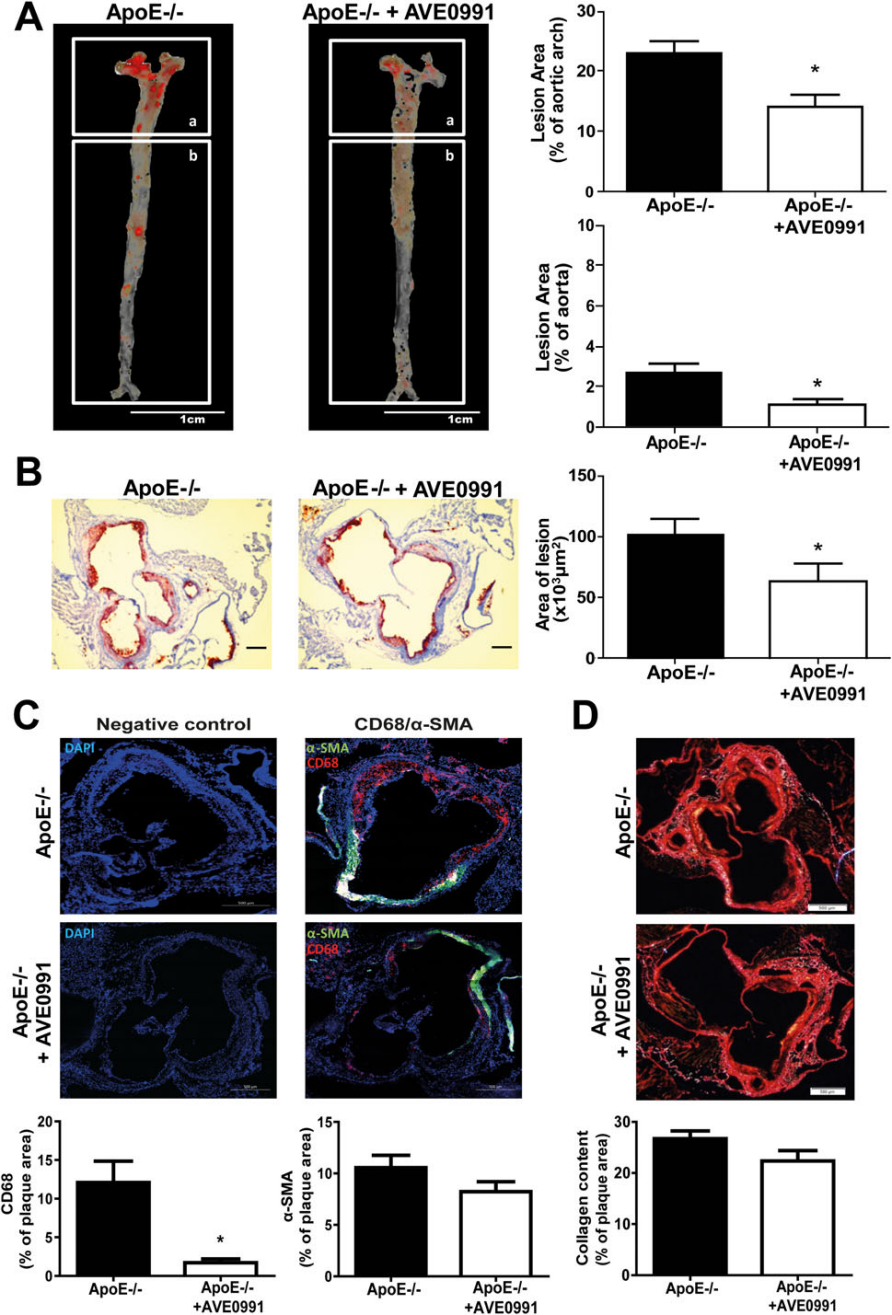
AVE0991 decreases atherosclerotic plaque development and macrophage infiltration, but not collagen content in plaques from ApoE−/− mice. (A) Oil Red O–stained ‘*en face’* preparation of aortas (scale bar = 1 cm, *n* = 5), and (B) Aortic sinus sections stained with Oil‐Red‐O obtained from 24‐week‐old ApoE−/− and ApoE−/− treated with AVE0991 (0.58 μmol·kg^−1^ day^−1^) (scale bar = 300 μm, *n* = 5). (C) Effect of 3 months AVE0991 treatment on plaque smooth muscle cell and macrophage content. Results are expressed as percentage of total plaque cells positive for α‐SMA (green) or CD68 (red) (bar = 500 μm) (*n* = 5). (D) Collagen staining of aortic root sections. Sections were stained using Picrosirius Red stain and visualized under polarized light, (bar = 500 μm, *n* = 5); Bars represent means ± SEM; *P < 0.05, significantly different from ApoE−/−.

ApoE−/− mice developed spontaneous plaque formation in both aortic sinus and in the remaining segments of aorta (in *en‐face* preparations). At the 24th week of age on chow diet, plaque was most evident in the aortic arch and sinus, with the descending aorta exhibiting moderate plaque formation (Figure 1A and B).

Oral administration (in the diet) of the Ang‐(1–7) mimetic, AVE0991, resulted in a significant reduction of spontaneous atherosclerosis development, demonstrated by Oil‐Red‐O staining in both aortic sinus and in *en‐face* preparations of the aortic arch and descending aorta at 24 weeks of age in ApoE−/− mice. In the aortic sinus, this protective effect was associated with dramatic reduction of macrophage content (Figure 1C), while smooth muscle cell and collagen content remained unaltered (Figure 1C and D), indicating significant anti‐inflammatory effect. This may be an important indicator of increased plaque stability after AVE0991 treatment.

### Early atherosclerosis is associated with significant perivascular leukocyte infiltration in ApoE−/− mice

**Figure 2:**
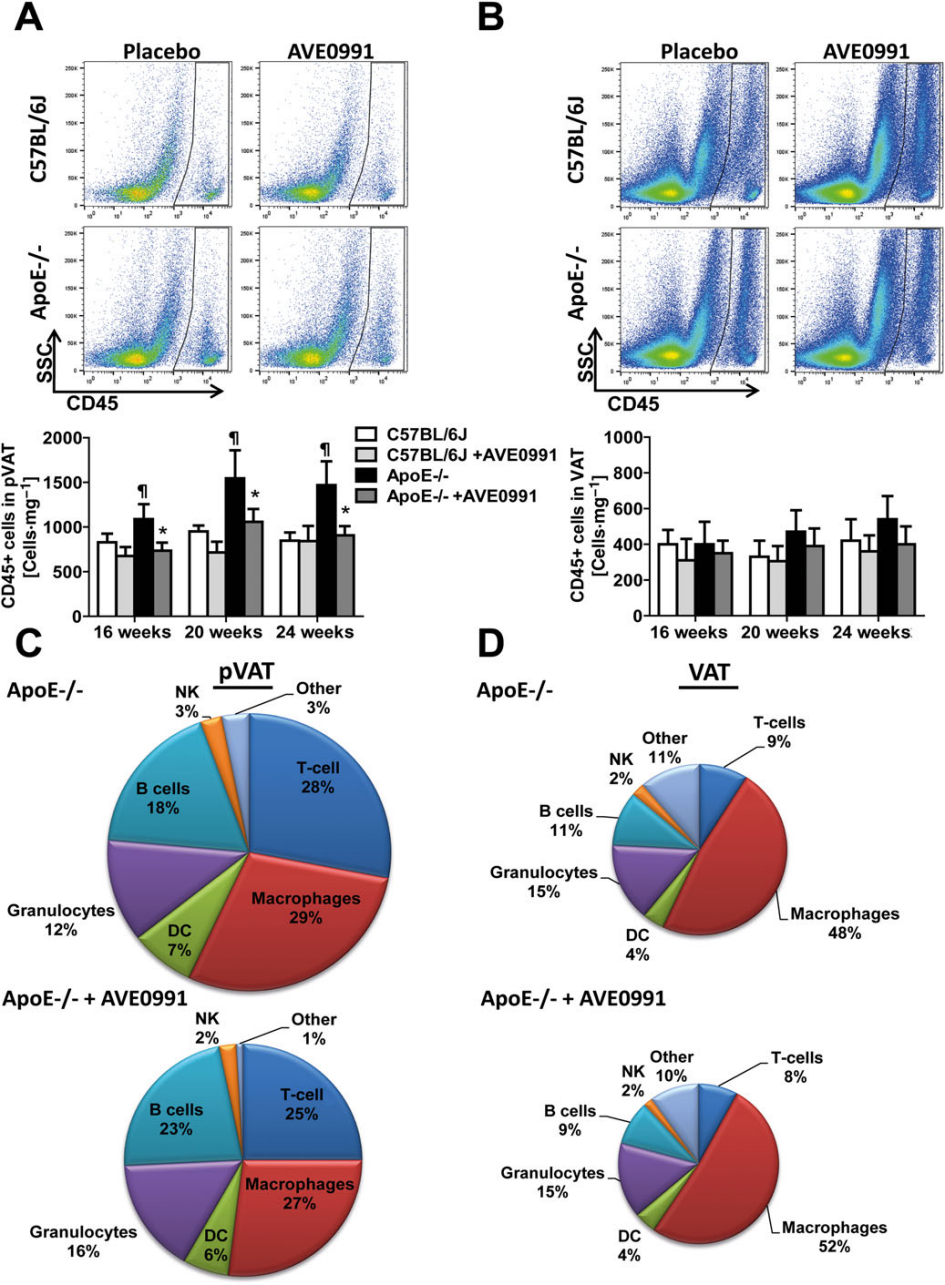
AVE0991 decreases leukocyte infiltration in PVAT, but not visceral AT in ApoE−/− mice at 16, 20 and 24 weeks of age. (A) Flow cytometric analysis of total CD45+ leukocytes in the vascular stromal fraction isolated from PVAT of ApoE−/− and C57BL/6J mice treated with placebo or AVE0991. Representative examples are shown (above) and (below) as means ± SEM (*n* = 12 each group). (B) Representative flow cytometric analysis of leukocytes in vascular stromal fraction isolated from visceral fat (VAT). Absolute number of CD45^+^ total leukocyte content in VAT compartment are expressed as means ± SEM (*n* = 12 each). (C) Pie‐charts describing % composition of subpopulations of leukocytes infiltrating PVAT and in (D), leukocytes in VAT**,** from 24‐week‐old ApoE−/− mice fed placebo and after treatment with AVE0991. Leukocyte subpopulations were identified as described in Methods. ^¶^ *P* < 0.05, significantly different from C57BL/6J mice; ^*^ *P* < 0.05, significant effects of AVE0991.

To study early vascular events during the development of atherosclerotic plaques, we focused on the descending aorta of 16, 20 and 24‐week‐old ApoE−/− mice on chow diet. We found that the total lesion, assessed by Oil‐Red‐O staining, was still low at 24 weeks of age (Figure 1A). At this stage, a moderate degree of impairment of endothelium dependent NO‐bioavailability in ApoE−/− mice was present (characterized by a shift in EC_50_ of relaxation to ACh), while at 16 weeks, no such features of dysfunction were observed (Supporting Information Fig. S2A and B). Interestingly, we also did not find differences in the leukocyte infiltration into the wall of the descending aorta, measured using flow cytometry to detect CD45+ cells, at either 16 or 24 weeks of age in ApoE−/− mice, compared with C57BL/6J controls (Supporting Information Fig. S2C). On the contrary, significantly increased leukocyte infiltration was detected, using this method, in the PVAT of ApoE−/− mice at 16 and 24 weeks of age, indicating that PVAT inflammation is an early feature of atherosclerotic vascular dysfunction (Supporting Information Fig. S2D). Importantly, such increased leukocyte infiltration was specifically observed in the PVAT of ApoE−/− mice, but not in typical visceral (epididymal) AT (Figure 2A and B).

### AVE0991 inhibits perivascular leukocyte infiltration in atherosclerosis

**Figure 3:**
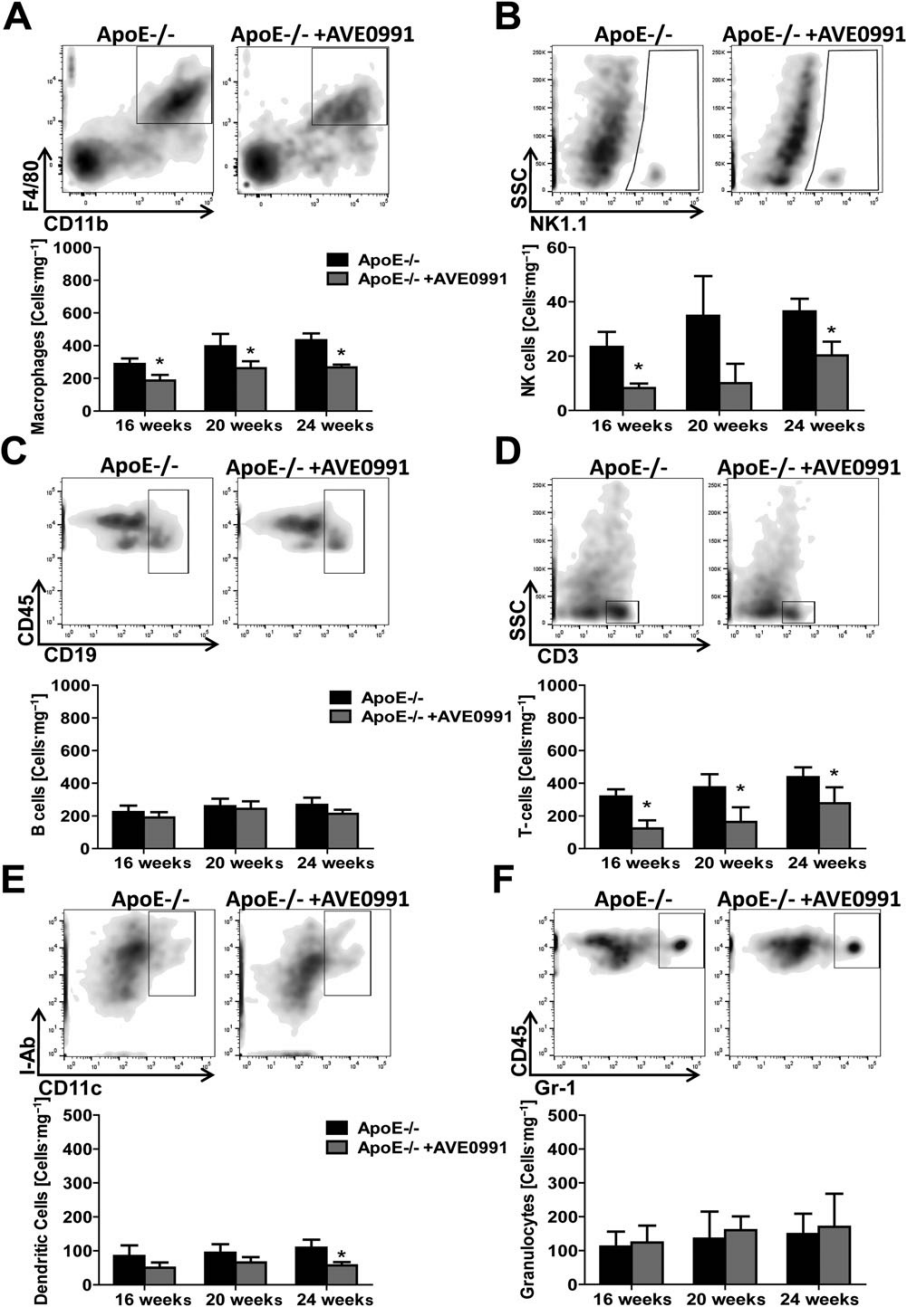
AVE0991 treatment decreases macrophage, NK cell, T‐cell and dendritic cell infiltration into PVAT in ApoE−/− mice. Representative flow cytometric analysis (24 weeks old) and average values of major leukocyte subpopulations in vascular stromal fraction isolated from PVAT of ApoE−/− and ApoE−/− mice treated with AVE0991, aged 16, 20 and 24 weeks. Data shown are means ± SEM. Graphs show the effects of AVE0991 treatment on content of (A) F4/80 + CD11b + macrophages (*n* = 12), (B) NK1.1+ NK cells (*n* = 6), (C) CD19+ B cells (n‐10), (D) CD3+ T‐cells (*n* = 12), (E) CD11c+ I‐Ab + dendritic cells (*n* = 6), and (F) Gr‐1+ granulocytes (*n* = 6) in PVAT. ^*^ *P* < 0.05, significant effects of AVE0991.

Next, we investigated the effects of oral treatment with AVE0991 on the PVAT leukocytes (as CD45+ cells). AVE0991 in the diet inhibited the PVAT content of CD45+ leukocytes, in 16, 20 and 24‐week‐old ApoE−/− mice (Figure 2A). In contrast, no effect was observed on leukocyte content in wild type mice (Figure 2A) or in epididymal visceral fat in either WT or ApoE−/− mice (Figure 2B and D) indicating specificity for inflammation in PVAT. Detailed analysis of leukocyte content revealed that the Ang‐(1–7) mimetic had the most effect on the reduction of macrophages, T‐cells and and natural killer (NK) cells in the PVAT, but not in epididymal visceral fat (Figures 2C–D and 3A–D). Importantly these inhibitory effects were most evident at 16 weeks of age in the ApoE−/− mice (Figure 3). The PVAT from ApoE−/− and C57BL/6J mice show similar gene expression for the Mas receptor (Supporting Information Table S4). Mas receptors are also expressed in the human monocyte cell line (THP‐1) (Supporting Information Table S4), indicating that moncytes can constitute an important target for AVE0991.

### AVE0991 inhibits chemokine and cytokine expression in PVAT in ApoE−/− mice

One of the key mechanisms for reduced macrophage and T‐cell perivascular infiltration in ApoE−/− mice may result from alteration of chemokines and adhesion molecules in the PVAT. Expression of key chemokines previously linked to atherosclerosis, including CCL2 and CXCL10 as well as CCL5, was significantly attenuated by AVE0991 treatment, in the PVAT of ApoE−/− mice, while no change was seen in the aortic wall itself (Table 1). This was associated with decreased expression of the adhesion molecule, ICAM‐1, in both PVAT and aorta (Table 1), which in concert with changes in chemokine expression may provide a mechanism for decreased recruitment of immune cells in early atherosclerosis. Importantly, key PVAT cytokines such as TNF‐α and IL‐6 were also significantly attenuated in AVE0991‐treated ApoE−/− mice (Table 1).

**Table 1:** Effects of AVE0991 on expression of mRNA for selected pro‐inflammatory cytokines, chemokines adhesion molecule in PVAT and aorta from ApoE−/− mice.

	Aorta		PVAT	
Gene	Fold change after AVE0991 treatment	*P* value	Fold change after AVE0991 treatment	*P* value
*IL6*	0.84	0.37	0.30	<0.05
*TNF‐α*	0.68	0.26	0.53	<0.05
*CXCL10*	0.81	0.33	0.24	<0.01
*CCL2*	0.52	0.22	0.38	<0.01
*CCL5*	0.88	0.42	0.65	<0.05
*CXCR3*	0.96	0.59	0.73	0.10
*ICAM‐1*	0.76	<0.05	0.60	<0.05

The mRNA levels of key cytokines known to play a role in atherogenesis and chemotaxis were analysed from PVAT and aorta from 24 weeks of age ApoE−/− mice and ApoE−/− mice treated with AVE0991 for 3 months. Results are shown as a fold change of mRNA expression from ApoE−/− mice after AVE0991 treatment to ApoE−/− mice itself. mRNA abundance was measured by quantitative real‐time PCR corrected for the mean *S18* mRNA expression. (*n* = 6 per group)

### AVE0991 affects macrophage polarization in PVAT in early atherosclerosis

**Figure 4:**
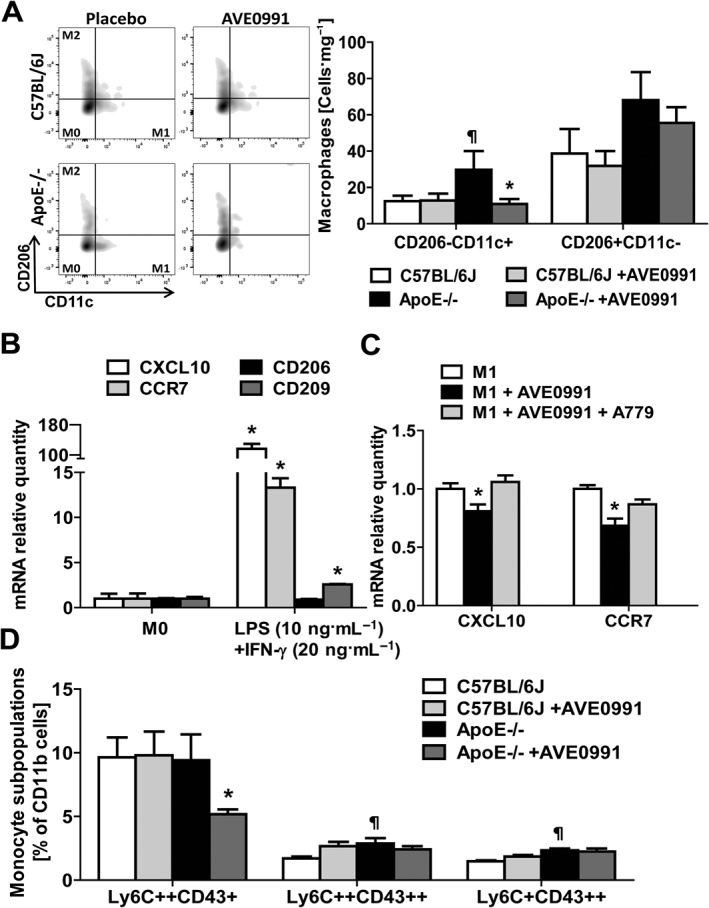
AVE0991 decreases M1 but not M2 macrophage content in PVAT in ApoE−/− mice and decreases M1 type differentiation of THP‐1 monocytes *in vitro*. (A) Flow cytometric analysis of M1/M2 macrophage polarization in PVAT isolated from C57BL/6J mice, C57BL/6J mice treated with AVE0991, ApoE−/− mice, and ApoE−/− mice treated with AVE0991 at 24 weeks of age. Effect of AVE0991 treatment on macrophages M1 and M2 content in PVAT expressed as cells per mg of tissue (mean ± SEM; *n* = 6 each); ^¶^ *P* < 0.05, significantly different from C57BL/6 J; ^*^ *P* < 0.05, significant effects of AVE0991. (B) Expression of mRNA marker genes for M1 macrophages (CCR7, CXCL10) and M2 macrophages (CD206, CD209) in cell culture of THP‐1 cells stimulated by LPS (10 ng·mL^‐1^) and IFN‐γ (20 ng·mL^‐1^) for 24 h. ^*^ *P* < 0.05, significantly different from M0. (C) Effect of AVE0991 (1 μM) treatment on M1 marker gene (CXCL10 and CCR7) mRNA expression after M0 macrophage polarization to M1 macrophages. A779 (5 μM) was used to study the role of Mas receptors in this process (*n* = 7); *P* < 0.05, significant effects of AVE0991. (D) AVE0991 decreases Ly6C++CD43+ monocytes in ApoE−/− mice. Monocyte subpopulations (Ly6C++CD43+, Ly6C++CD43++, Ly6C + CD43++) in PBMC of 24‐week‐old C57BL/6J and ApoE−/− mice upon treatment with AVE0991; ^¶^ *P* < 0.05, versus C57BL/6J; ^*^ *P* < 0.05, significant effects of AVE0991; (*n* = 6 per group).

ApoE−/− mice were characterized by increased M1 macrophage content in PVAT as observed by flow cytometric determination of CD206 and CD11c surface expression on macrophages (Figure 4A). AVE0991 reduced the M1 macrophage population in ApoE−/− mice while having no effect on M2 macrophage content (Figure 4A). In order to further evaluate direct effects of AVE0991 on macrophage polarization, we used THP‐1 cells in culture during *in vitro* differentiation to M1 macrophages following stimulation with LPS and IFN‐γ, as described in the Methods section (Figure 4B). This confirmed that AVE0991 inhibited the expression of mRNA for M1 markers (CXCL10 and CCR7) during differentiation (Figure 4C). Importantly this effect on M1 differentiation was reversed by incubation with the Mas receptor antagonist A779 before exposure to AVE0991(Figure 4C).

### AVE0991 affects peripheral blood monocyte subsets

*In vivo* administration of AVE0991 resulted in a shift in peripheral blood monocyte subsets as determined by Ly6C and CD43 marker expression. In particular, content of Ly6C++Cd43+ pro‐inflammatory monocytes were reduced (Figure 4D).

### AVE0991 inhibits monocyte/macrophage pro‐inflammatory activation *in vitro*

**Figure 5:**
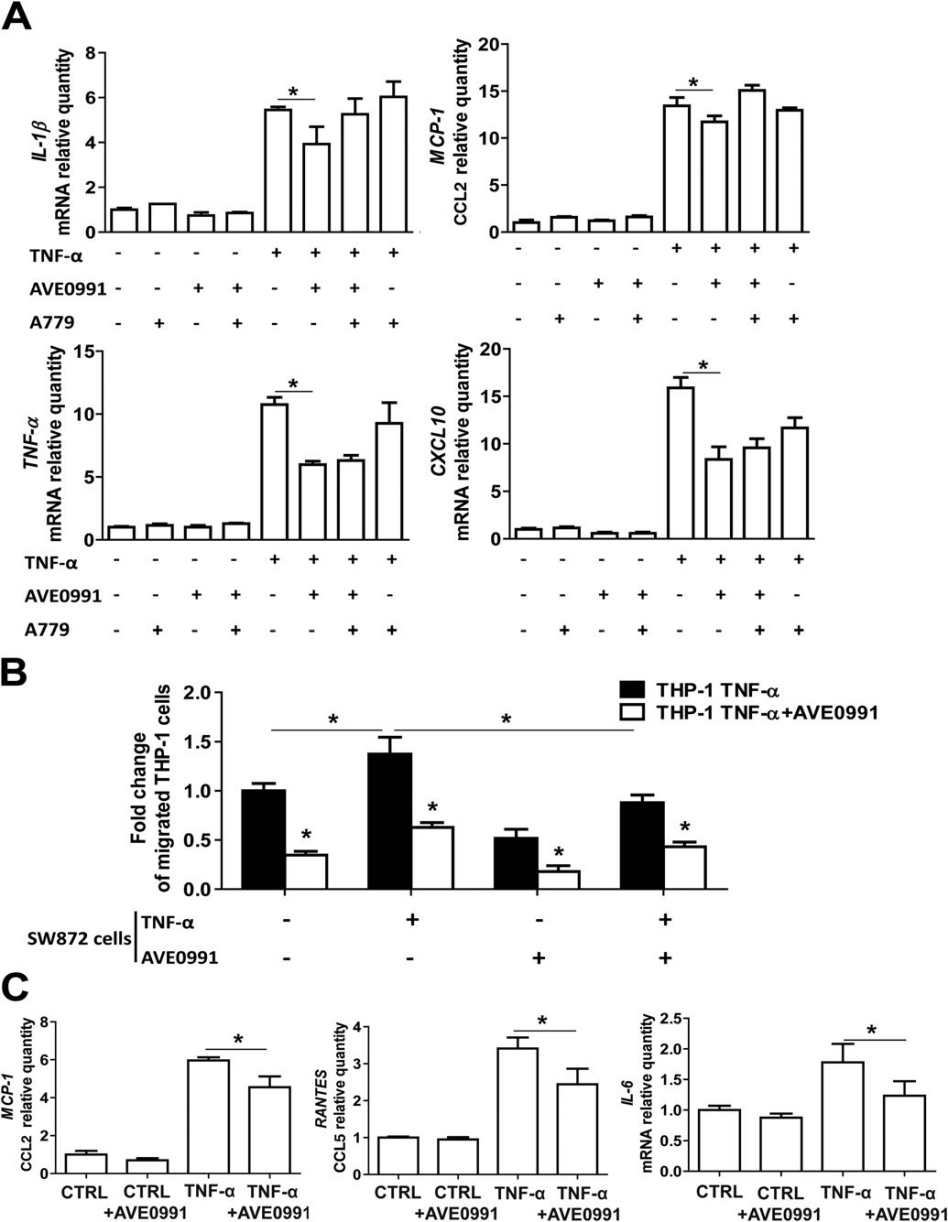
Effect of AVE0991 on TNF‐α activated THP‐1 cytokine and chemokine mRNA expression, chemotaxis towards human adipocytes and chemokine expression in human adipocytes. (A) Expression of selected pro‐inflammatory mRNA cytokines and chemokines in THP‐1 cells stimulated with TNF‐α (10 ng·mL^‐1^) in presence of AVE0991 (1 μM) and/or the Mas receptor antagonist A779 (5 μM) (*n* = 6). (B) Chemotactic properties of THP‐1 cells stimulated with TNF‐α to supernatants from SW872 adipocyte cultures, stimulated with TNF‐α in absence or presence of AVE0991 (1 μM). Cell chemotaxis was analysed and expressed as a fold change of the number of cells passing through 8 μm pores under different conditions, compared with THP‐1 cells stimulated with TNF‐α (10 ng mL^‐1^) and with supernatants from native SW872 adipocytes without additional treatment. Before testing chemotaxis, the THP‐1 cells were stimulated for 6 h with TNF‐α or stimulated with TNF‐α for 6 h and then incubated with AVE0991 (1 μM) for 18 h (*n* = 5). (C) Expression of pro‐inflammatory CCL2, CCL5 and IL6 mRNA in SW872 cells differentiated to adipocytes and stimulated with TNF‐α (10 ng cm^−3^) or saline (CTRL), with or without AVE0991 (1 μM) (*n* = 6); ^*^ *P* < 0.05, significantly different as indicated; *t*‐ test.

To further investigate the anti‐inflammatory mechanisms of AVE0991, we studied the effects of 1 μM AVE0991 on TNF‐α stimulated phenotypic changes of THP‐1 monocytes in culture. We observed that AVE0991‐inhibited TNF‐α induced THP‐1 cell activation characterized by increased expression of mNA for IL‐1β, CCL2 and CXCL10 (Figure 5A). These changes were partly reversed by the Mas receptor antagonist A779 (Figure 5A).

### AVE0991 inhibits monocyte chemotaxis to adipocytes *in vitro*

To address the effects of AVE0991 on monocyte chemotaxis, we performed a chemotaxis assay using Boyden chambers to assess the effects of AVE0991 treatment on chemotaxis of TNF‐α activated THP‐1 cells towards SW872 adipocyte supernatants (in the bottom chamber). These studies showed that pretreatment of THP‐1 cells with AVE0991 resulted in a significant reduction of chemotaxis towards the supernatants of both native and TNF‐α activated SW872 human adipocytes (Figure 5B). Pretreatment of SW872 cells with AVE0991 resulted in similar inhibition of chemotaxis, but no additive effect was observed.

### AVE0991 alters chemokine expression in human adipocytes in culture

To investigate the effects of AVE0991 on adipocytes, we used cultures of TNF‐α stimulated SW872 human adipocytes. These experiments showed that AVE0991 inhibited expression of mRNA for CCL2, CCL5, and IL‐6 (Figure 5C). Similar experiments, performed in human fibroblasts and VSMCs, did not show significant effects of AVE0991 (Supporting Information Figure S3), indicating that adipocytes and immune cells constitute primary targets for anti‐inflammatory actions of AVE0991.

## Discussion

While it is widely accepted that atherosclerosis is an immune mediated disease (Ait‐Oufella *et al.*, 2011; Ketelhuth and Hansson, 2016; Libby *et al.*, 2011; Ridker and Luscher, 2014; Weber and Noels, 2011), none of the clinically accepted pharmacological treatments directly target the development of vascular inflammation in atherosclerosis (Matusik *et al.*, 2012). Most of our knowledge regarding inflammation and immunity in cardiovascular disease is derived from studies of advanced atherosclerotic plaques, and little is known regarding the early stages of inflammation in atherosclerosis. Accordingly, in the present study, using ApoE‐/‐ mice fed normal chow, we have found that the increased leukocyte infiltration in the perivascular tissue was greater than that in the vessel wall, measured by flow cytometry. Risk factors for atherosclerosis such as hypertension, hyperlipidemia or diabetes are known to promote perivascular inflammation (Galkina *et al.*, 2006; Galkina and Ley, 2009; Guzik *et al.*, 2013; Guzik *et al.*, 2007b; Sagan *et al.*, 2012; Vu *et al.*, 2014). The renin‐angiotensin‐system (RAS), and in particular angiotensin II, is key in increasing perivascular recruitment of leukocytes (Guzik *et al.*, 2007a; Mikolajczyk *et al.*, 2016), acting through an increase of chemokine expression in the PVAT. CCL5 or CCL2 are increased in early atherosclerosis (Podolec *et al.*, 2016). The RAS exerts its pro‐atherosclerotic effects also through T cell, B cell and monocyte/macrophage activation (Hoch *et al.*, 2009; Kirabo *et al.*, 2014). We postulated that stimulation of the alternative axis within the RAS, related to Ang‐(1‐7) signalling, could provide a counter‐regulatory, anti‐inflammatory effect in early atherosclerosis (Tesanovic *et al.*, 2010). Both inflammatory cells as well as perivascular adipocytes and fibroblasts express Mas receptors for Ang‐(1‐7) (Rubio‐Ruiz *et al.*, 2014; Simoes e Silva *et al.*, 2013). Mas receptor expression was higher in PVAT than in other areas of aorta and it was not significantly increased in ApoE‐/‐ mice, in contrast to reports of the mouse penis (Fraga‐Silva *et al.*, 2013),

Taking all these results into consideration, we used orally active, selective Mas receptor agonist AVE0991 to stimulate the Ang‐(1‐7) protective axis in ApoE‐/‐ mice, and studied its effects on atherosclerosis and vascular inflammation. We found significant anti‐atherosclerotic properties in ApoE‐/‐ mice (Jawien *et al.*, 2012a) and extended these observations to show that AVE0991 affects atherosclerosis development at early stages of plaque formation, in the descending aorta in young ApoE‐/‐ mice. In advanced plaques, we also showed that AVE0991 reduced plaque macrophages, without changing collagen content, thus beneficially affecting plaque stability. This is in line with observations that AVE0991 can affect co‐stimulatory molecules expression on CD11c+ cells in mouse spleen in atherosclerosis (Jawien *et al.*, 2012a). To assess the anti‐inflammatory effects of AVE0991 during the early stages of atherosclerosis, we focused on characterization of vascular and perivascular immune cells between the 16^th^ and the 24^th^ week of age in chow‐fed ApoE‐/‐ mice. At week 16, plaques begin to develop in the descending aorta but they constitute <3% of its total surface (Elhage *et al.*, 2004) and no significant NO‐dependent endothelial dysfunction is detected at this stage (Bonthu *et al.*, 1997; Fransen *et al.*, 2008; Jawien *et al.*, 2004). We found that endothelial dysfunction appeared in the 24^th^ week, at a time when the plaque burden in the descending aorta starts to exceed 4‐5% of the total surface. This is important, as endothelial dysfunction is considered to underlie atherosclerosis development in ApoE‐/‐ mice (Laursen *et al.*, 2001). In the present study, we observed that in the absence of Western diet, PVAT or adventitial inflammation and infiltration with macrophages and T cells precedes not only significant atherosclerotic plaque development, but also the impairment of endothelium‐dependent NO bioavailability. Importantly, a similar leukocyte increase was not observed in a typical visceral fat tissue (epididymal AT). The composition of the leukocyte subpopulations in PVAT was similar to reports in advanced atherosclerosis (Galkina *et al.*, 2006) and in hypertension (Guzik *et al.*, 2007a; Mikolajczyk *et al.*, 2016). Interestingly the main difference in PVAT infiltration between these two conditions is in relative macrophage and T cell content (macrophages predominate in atherosclerosis while T cells predominate in hypertension). AVE0991 reduced perivascular infiltration of macrophages, T cells and NK cells while infiltration of B cells, granulocytes and dendritic cells was unaffected. Mas receptors have been identified on majority of immune cells including macrophages, dendritic cells, T cells and Mas receptor deficiency exacerbates systemic inflammation (Oliveira‐Lima *et al.*, 2015). Lack of effect of AVE0991 on PVAT leukocyte infiltration in the control group may also indicate different mechanisms being involved in the regulation of leukocyte accumulation in control and atherosclerotic conditions. Ang‐(1‐7) and the synthetic Mas receptor agonist AVE 0991 have been effective in a number of inflammatory models, such as arthritis (da Silveira *et al.*, 2010), asthmatic lung inflammation (Rodrigues‐Machado *et al.*, 2013) and renal inflammation (Silveira *et al.*, 2013).

The mechanisms of the anti‐atherosclerotic effect of AVE0991 was related to the reduction of chemokine (CCL2, CCL5, CXCL10) expression in PVAT, as well as through direct actions on monocytes/macrophages, inhibiting their activation, characterized by IL‐1β, TNF‐α, CCL2 and CXCL10, and differentiation to the M1 phenotype. AVE0991 inhibited migration of THP‐1 monocytes towards supernatants of activated adipocytes (SW872). Thus, through *in vivo* and *in vitro* studies, we have demonstrated that the Mas receptor agonist AVE0991 exhibits anti‐inflammatory properties affecting monocyte/macrophage differentiation and recruitment to the perivascular space at early stages of atherosclerosis in ApoE‐/‐ mice.

Our demonstration of the Mas receptor‐dependent inhibition of M1 macrophage differentiation by AVE0991 may extend the potential usefulness of this compound to a number of conditions in which M1 macrophages play a key role (Hammer *et al.*, 2016). This includes pulmonary remodelling, inflammation and right ventricular hypertrophy in models of allergic asthma (Rodrigues‐Machado *et al.*, 2013), as well as its protective effects in stroke (Lee *et al.*, 2015) or liver cirrhosis (Klein *et al.*, 2015). Mechanisms of the vasoprotective effects of AVE0991 include inhibition of oxidative stress (Ma *et al.*, 2016), stimulation of eNOS activation and NO production (Pawlik *et al.*, 2016). It also exerts effects in the CNS, decreasing sympathetic outflow and renal effects leading to changes in the renal proteome, in trems of antioxidant enzymes, apoptosis regulators, inflammatory factors and metabolic enzymes (Suski *et al.*, 2013). AVE0991 also inhibits p22phox(Jawien *et al.*, 2012a) and Nox2 and Nox4 NADPH oxidases (Ma *et al.*, 2016) and thus can change the pro‐ and anti‐oxidant balance, indirectly affecting vascular inflammation (Guzik *et al.*, 2005).

While immune cell infiltration has been shown in fully developed human and mouse atherosclerotic plaques (Aubry *et al.*, 2004), using flow cytometry, we did not detect significant numbers of leukocytes in aortic intima and media, in 16‐24 week old ApoE‐/‐ mice. This finding could indicate a re‐distribution of immune cells in the vessel during development of atherosclerosis (Chalmers *et al.*, 2015). Importantly, the overall numbers of leukocytes detected in the aortic intima and media are 5‐fold lower than those in the adventitia and in PVAT. This adventitial and periadventitial immune cell presence remains throughout the course of atherosclerosis (Galkina *et al.*, 2006; Galkina and Ley, 2009). Depending on the stage of atherosclerosis, accumulations of these cells, consisting predominantly of T cells and B cells and to a lesser extent of other immune cells like dendritic cells or macrophages, termed tertiary lymphoid organs, may be pro‐ or anti‐atherogenic (Clement *et al.*, 2015; Hu *et al.*, 2015; Srikakulapu *et al.*, 2016). While the majority of studies looking at perivascular leukocyte infiltration in atherosclerosis have focused on adventitia, inflammation in the PVAT is evident from the earliest stages. The mechanisms linking PVAT inflammation to atherosclerosis may be diverse and may include chemotactic migration of PVAT immune cells into adventitia and plaques, with release of cytokines which can alter vascular function (Mikolajczyk *et al.*, 2016). Inflammation may also alter release of adipokines and adipocyte derived relaxing factor from adipocytes (Antonopoulos *et al.*, 2015; Woodward *et al.*, 2016). All of these mechanisms can initiate and propagate plaque formation and plaque‐specific inflammation (Libby *et al.*, 2011; Weber *et al.*, 2011; Back and Hansson, 2015).

It is important to note that, in our studies, female mice were used. This is a classical model in which immuno‐pathogenesis of atherosclerosis has been described (Caligiuri *et al.*, 1999), but both the RAS and inflammation differ between male and female mice, including higher renal Ang‐(1‐7) concentration (Zimmerman *et al.*, 2015). Interestingly, we did not find (by mass spectrometry) differences between the wild‐type (C57BL/6J) and ApoE‐/‐ mice in Ang‐(1‐7), angiotensin‐1‐9 and angiotensin II levels in PVAT (data not shown).

Our study does not allow for unequivocal explanation of the receptor‐mediated mechanisms. While several studies shown high selectivity of AVE0991 towards Mas receptors (Santos *et al.*, 2003), in selected conditions, effects could be mediated partly through angiotensin AT_1_ or AT_2_ receptors (Tesanovic *et al.*, 2010; Bosnyak *et al.*, 2011). Partial reversal of anti‐inflammatory mechanisms by the Mas antagonist A779, in our *in vitro* studies, indicates a need for further studies using a combination of Mas receptor and AT_2_ receptor blockade.

In summary, our results may provide a key mechanism for the early anti‐atherosclerotic effects of AVE0991. This also suggests that AVE0991 could provide a valuable alternative to typical immunosuppressants in vascular disease as it acts simultaneously on the vasculature and on immune/inflammatory cells. Such treatment could be more widely accepted by the cardiovascular community while its effects on immune and inflammatory mechanisms of atherosclerosis are clear.

## Author contributions

D.S.S. designed and conducted the majority of experiments, analysed data, prepared figures and co‐wrote the manuscript; R.N. performed vascular and flow cytometry experiments, analysed the data; T.P.M. designed and analysed immunological experiments; M.S. participated in data analysis; F.J.R. designed *in vitro* experiments; revised critically for intellectual contribution to the manuscript; A.C.M. made intellectual contributions to the manuscript; J.J. and R.O. helped with design of *in vivo* experiments; R.M.T. supervised experiments, revised critically for intellectual contribution to the manuscript; R.K. provided critical comments on manuscript; M.C.G. contributed reagents and provided comments on manuscript; T.J.G. conceived, designed and supervised experiments and wrote the manuscript.

## Conflict of interest

The authors declare no conflicts of interest.

## Declaration of transparency and scientific rigour

This Declaration acknowledges that this paper adheres to the principles for transparent reporting and scientific rigour of preclinical research recommended by funding agencies, publishers and other organisations engaged with supporting research.

## Supporting information


**Table S1** List of TaqMan® probes.
**Table S2** List of human primers used for SybrGreen qPCR.
**Table S3** Flow cytometry antibodies used.
**Table S4** Mas receptor mRNA expression in PVAT WT and ApoE‐/‐ (*n*=6‐10), in aorta and PVAT from ApoE‐/‐ (*n*=6) and upon THP‐1 cell activation (*n*=5).
**Figure S1** (A) Experimental design and AVE0991 administration. Fifty four 12 weeks of age C57BL/6J female mice and fifty 12 weeks of age ApoE‐/‐ mice were put on chow diet. To observe effect of the Mas1 receptor agonist, AVE0991 (0.58μmol kg^‐1^ body weight day^‐1^; Sanofi‐Aventis, Germany) was added to food (Soest, Germany) and twenty four C57BL/6J female mice and fifty four ApoE‐/‐ mice were feed. To observe progress of atherosclerosis and inflammatory state development, mice were killed at 16, 20 and 24 weeks of age. (B) Flow cytometric gating strategy. Leukocytes were gated by CD45 staining. From leukocytes, further sub‐populations of B cells (CD19+), dendritic cells (CD11c+I‐Ab+), granulocytes (Ly6c+Ly6g+), macrophages (F4/80+CD11b+), natural killer (NK) cells (NK1.1+) and T‐cells (CD3+) were identified. To demonstrate different states of macrophage polarization, we set up gates for M0 macrophages (defined as CD206‐CD11c‐), M1 macrophages (CD206‐CD11c+) and M2 macrophages (CD206+CD11c‐).
**Figure S2** At early stage of atherosclerosis in ApoE‐/‐ mice there is no impairment of endothelium‐dependent NO bioavailability and visible inflammation in aorta whereas increase of inflammatory cells infiltration is evident in perivascular adipose tissue. (A, in C57BL/6J and ApoE‐/‐ mice at (A) 16 weeks and (B) 24 weeks of age (*n*=5) (C, D) Representative flow cytometric analysis of leukocytes infiltration to (C) aorta and (D) periaortic adipose tissue in C57BL/6J and ApoE‐/‐ mice at 24 weeks of age. Leukocytes infiltration was calculated as a cell number per mg of tissue. *P<0.05 by T test.
**Figure S3** Effect of AVE0991 on TNF‐α activated human vascular smooth muscle cells (HVSMC) cytokine and chemokine mRNA expression. Expression of selected proinflammatory mRNA cytokines and chemokines in HVSM cells stimulated with TNF‐α (10ng cm^‐3^) in presence of AVE0991 (1μM) and/or Mas receptor antagonist A779 (5μM) (*n*=5). *P<0.05 vs CTRL (control) by T test.
