# Supplementary material for: Anti‐atherosclerotic effect of the angiotensin 1–7 mimetic AVE0991 is mediated by inhibition of perivascular and plaque inflammation in early atherosclerosis
Source: Br J Pharmacol. 2017 Feb 1;174(22):4055–69. doi: 10.1111/bph.13685 (PMC5659999; doi:10.1111/bph.13685)
Supplement: Supplementary file 1 — Table S1 List of TaqMan® probes. Table S2 List of human primers used for SybrGreen qPCR. Table S3 Flow cytometry antibodies used. Table S4 Mas receptor mRNA expression in PVAT WT and ApoE‐/‐ (n=6‐10), in aorta and PVAT from ApoE‐/‐ (n=6) and upon THP‐1 cell activation (n=5). Figure S1 (A) Experimental design and AVE0991 administration. Fifty four 12 weeks of age C57BL/6J female mice and fifty 12 weeks of age ApoE‐/‐ mice were put on chow diet. To observe effect of the Mas1 receptor agonist, AVE0991 (0.58μmol kg‐1 body weight day‐1; Sanofi‐Aventis, Germany) was added to food (Soest, Germany) and twenty four C57BL/6J female mice and fifty four ApoE‐/‐ mice were feed. To observe progress of atherosclerosis and inflammatory state development, mice were killed at 16, 20 and 24 weeks of age. (B) Flow cytometric gating strategy. Leukocytes were gated by CD45 staining. From leukocytes, further sub‐populations of B cells (CD19+), dendritic cells (CD11c+I‐Ab+), granulocytes (Ly6c+Ly6g+), macrophages (F4/80+CD11b+), natural killer (NK) cells (NK1.1+) and T‐cells (CD3+) were identified. To demonstrate different states of macrophage polarization, we set up gates for M0 macrophages (defined as CD206‐CD11c‐), M1 macrophages (CD206‐CD11c+) and M2 macrophages (CD206+CD11c‐). Figure S2 At early stage of atherosclerosis in ApoE‐/‐ mice there is no impairment of endothelium‐dependent NO bioavailability and visible inflammation in aorta whereas increase of inflammatory cells infiltration is evident in perivascular adipose tissue. (A, in C57BL/6J and ApoE‐/‐ mice at (A) 16 weeks and (B) 24 weeks of age (n=5) (C, D) Representative flow cytometric analysis of leukocytes infiltration to (C) aorta and (D) periaortic adipose tissue in C57BL/6J and ApoE‐/‐ mice at 24 weeks of age. Leukocytes infiltration was calculated as a cell number per mg of tissue. *P<0.05 by T test. Figure S3 Effect of AVE0991 on TNF‐α activated human vascular smooth muscle cells (HVSMC) cytokine and chemokine mRNA expre [file BPH-174-4055-s001.pdf]

Supplementary materials

Table S1. List of TaqMan® probes.

Gene	Catalogue number	Gene	Catalogue number
**Mouse**		**Human**	
Agtr1a	Mm00616371_m1	*AGTR1*	Hs00258938_m1
Agtr2	Mm01341373_m1	*AGTR2*	Hs02621316_s1
Mas1	Mm01313002_m	*MAS1*	Hs00267157_s1
Ccl5	Mm01302427_m1		
Ccl2	Mm00441242_m1		
Rn18s	Mm03928990_g1		
Icam1	Mm00516023_m1		
Cxcl10	Mm00445235_m1		
Cxcr3	Mm99999054_s1		
IL6	Mm00446190_m1		
Tnf-α	Mm00443258_m1		

Table S2. List of human primers used for SybrGreen qPCR.

Gene	Forward primer sequence (5'→3')	Reverse primer sequence (5'→3')
CCR7	TGGTGGTGGCTCTCCTTGTC	TGTGGTGTTGTCTCCGATGTAATC
MRC1	ACCTCACAAGTATCCACACCATC	CTTTCATCACCACACAATCCTC
CD209	TCAAGCAGTATTGGAACAGAGGA	CAGGAGGCTGCGGACTTTTT
CXCL10	ATTTGCTGCCTTATCTTTCTG	TCTCACCCTTCTTTTTCATTGTAG
IL1B	TCCAGGGACAGGATATGGAG	TCTTTCAACACGCAGGACAG
TNF	AGCCCATGTTGTAGCAAACC	TGAGGTACAGGCCCTCTGAT
MCP1	CCCCAGTCACCTGCTGTTAT	AGATCTCCTTGGCCACAATG

Table S3. Flow cytometry antibodies used

Antibody	Clone	Company
**anti-CD45 PerCP**	Clone 30-F11	BD Bioscience
**anti-CD45 V450**	Clone 30-F11	BD Bioscience
**anti-CD3e APC**	Clone 145-2C11	BD Bioscience
**anti-CD8a PerCP**	Clone 53-6.7	BD Bioscience
**anti-CD4 APC-H7**	Clone GK1.5	BD Bioscience
**anti-CD19 APC-H7**	Clone 1D3	BD Bioscience
**anti-I-A[b] FITC**	Clone AF6-120.1	BD Bioscience
**anti-CD11b PE**	Clone M1/70	BD Bioscience
**anti-CD11c APC**	Clone HL3	BD Bioscience
**anti-Gr1 APC**	Clone RB6-8C5	BD Bioscience
**anti-NK-1.1 APC**	Clone PK136	BD Bioscience
**anti-CD11b APC-Cy7**	Clone M1/70	BD Bioscience
**anti-CD11c PE**	Clone HL3	BD Bioscience
**anti-CD206 FITC**	Clone C068C2	BioLegend
**anti-F4/80 APC**	Clone BM8	eBioscience
**anti-Ly6C PE**	Clone AL-21	BD Bioscience
**anti-Ly6G PE-Cy7**	Clone 1A8	BD Bioscience
**anti-CD43 FITC**	Clone S7	BD Bioscience

Table S4. Mas receptor mRNA expression in pVAT WT and ApoE-/- (n=6-10), in aorta and pVAT from ApoE-/- (n=6) and upon THP-1 cell activation (n=5)

Gene	Description	Fold change	p value
Mas1	C57BL/6J	1.00	
	ApoE-/-	1.79	0.11
Mas1	ApoE-/- aorta	1.00	
	ApoE-/- pVAT	5.21	0.008
Mas1	THP-1	1.00	
	THP-1 + TNF-α	1.21	0.89

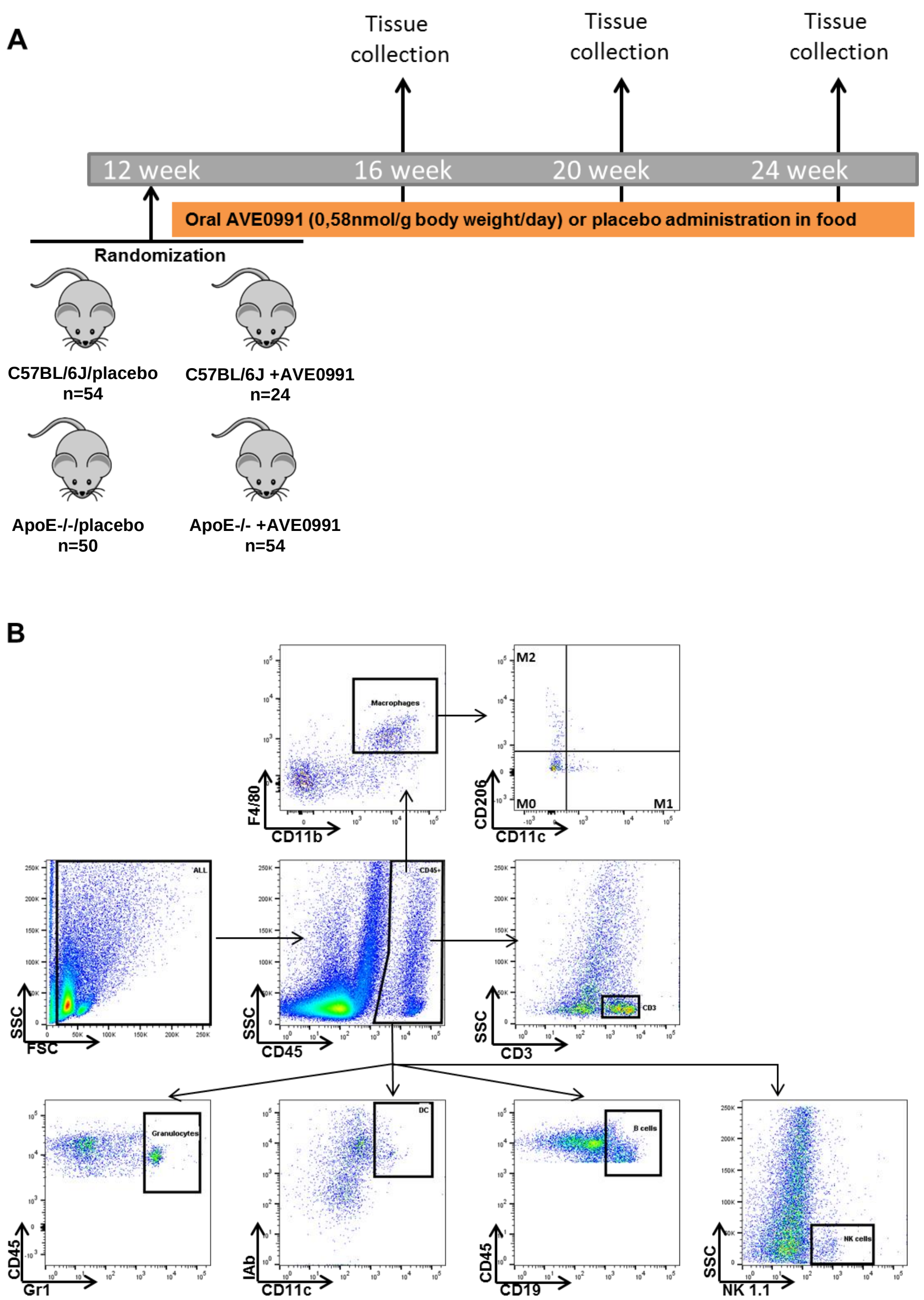

Figure Suppl. 1. (A) Experimental design and AVE0991 administration. Fifty four 12 weeks of age C57BL/6J female mice and fifty 12 weeks of age ApoE-/- mice were put on chow diet. To observe effect of Mas1 receptor agonist, AVE0991 ($0.58 \mu mol\ kg^{-1}$ body weight day^{-1}; Sanofi-Aventis, Germany) was added to food (Soest, Germany) and twenty four C57BL/6J female mice and fifty four ApoE-/- mice were feed. To observe progress of atherosclerosis and inflammatory state development, mice were sacrificed at 16, 20 and 24 weeks of age. **(B) Flow cytometric gating strategy.** Leukocytes were gated by CD45 staining. From leukocytes population B cells (CD19+), Dendritic Cells (CD11c+I-Ab+), Granulocytes (Ly6c+Ly6g+), macrophages (F4/80+CD11b+), NK cells (NK1.1+) and T cell (CD3+) were obtained. To distinguish macrophages polarization, gates for M0 macrophages (CD206-CD11c-), M1 macrophages (CD206-CD11c+) and M2 macrophages (CD206+CD11c-) were setup.

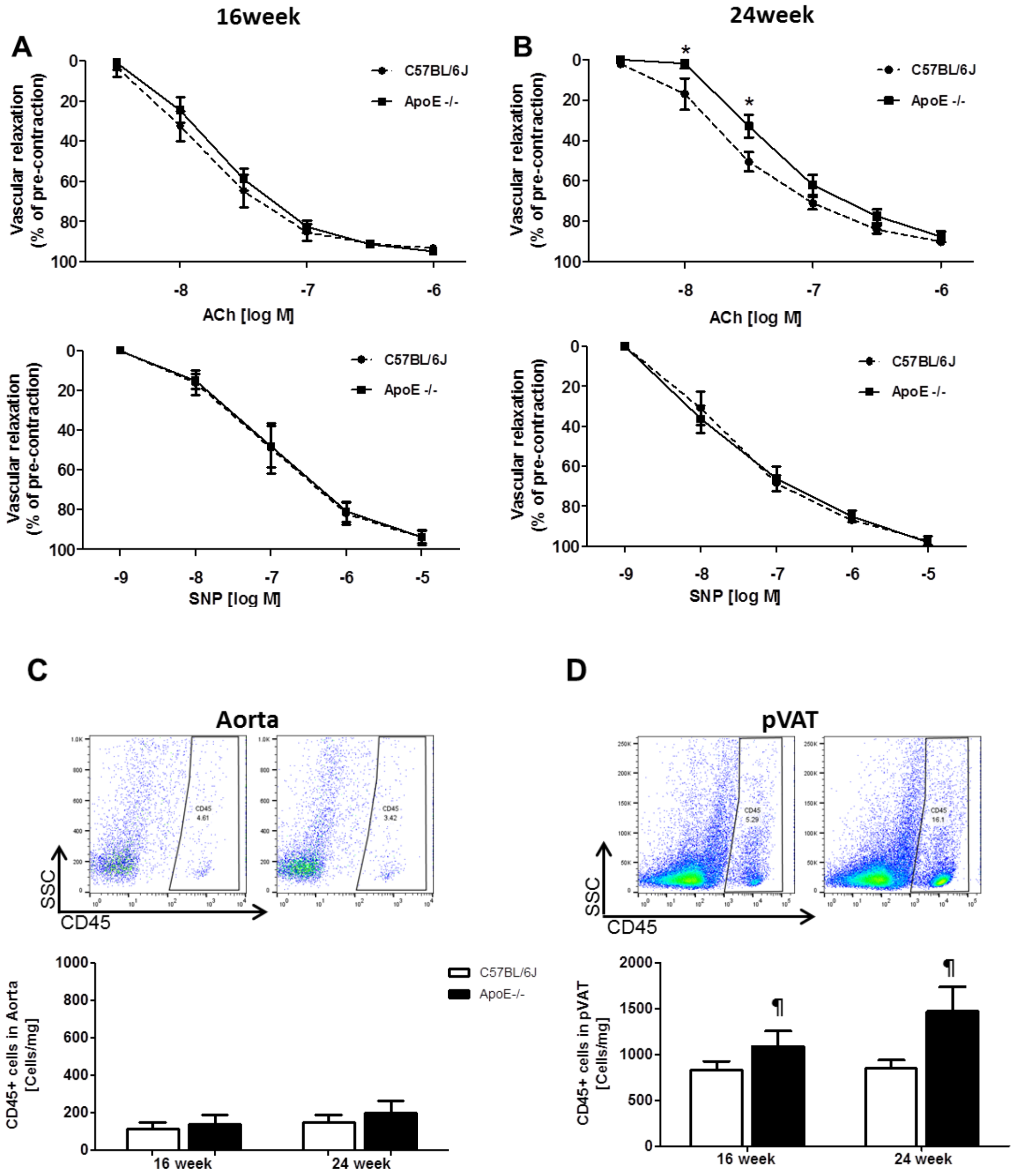

Figure Suppl. 2. At early stage of atherosclerosis in ApoE-/- mice there is no impairment of endothelium-dependent NO bioavailability and visible inflammation in aorta whereas increase of inflammatory cells infiltration is evident in perivascular adipose tissue. (A, B) Vascular relaxation endothelial dependent (ACh) and independent (SNP) were analysed in C57BL/6J and ApoE-/- mice at **(A)** 16 weeks and **(B)** 24 weeks of age (n=5) **(C, D)** Representative flow cytometric analysis of leukocytes infiltration to **(C)** aorta and **(D)** periaortic adipose tissue in C57BL/6J and ApoE-/- mice at 24 weeks of age. Leukocytes infiltration was calculated as a cell number per mg of tissue. *p<0.05 by T test.

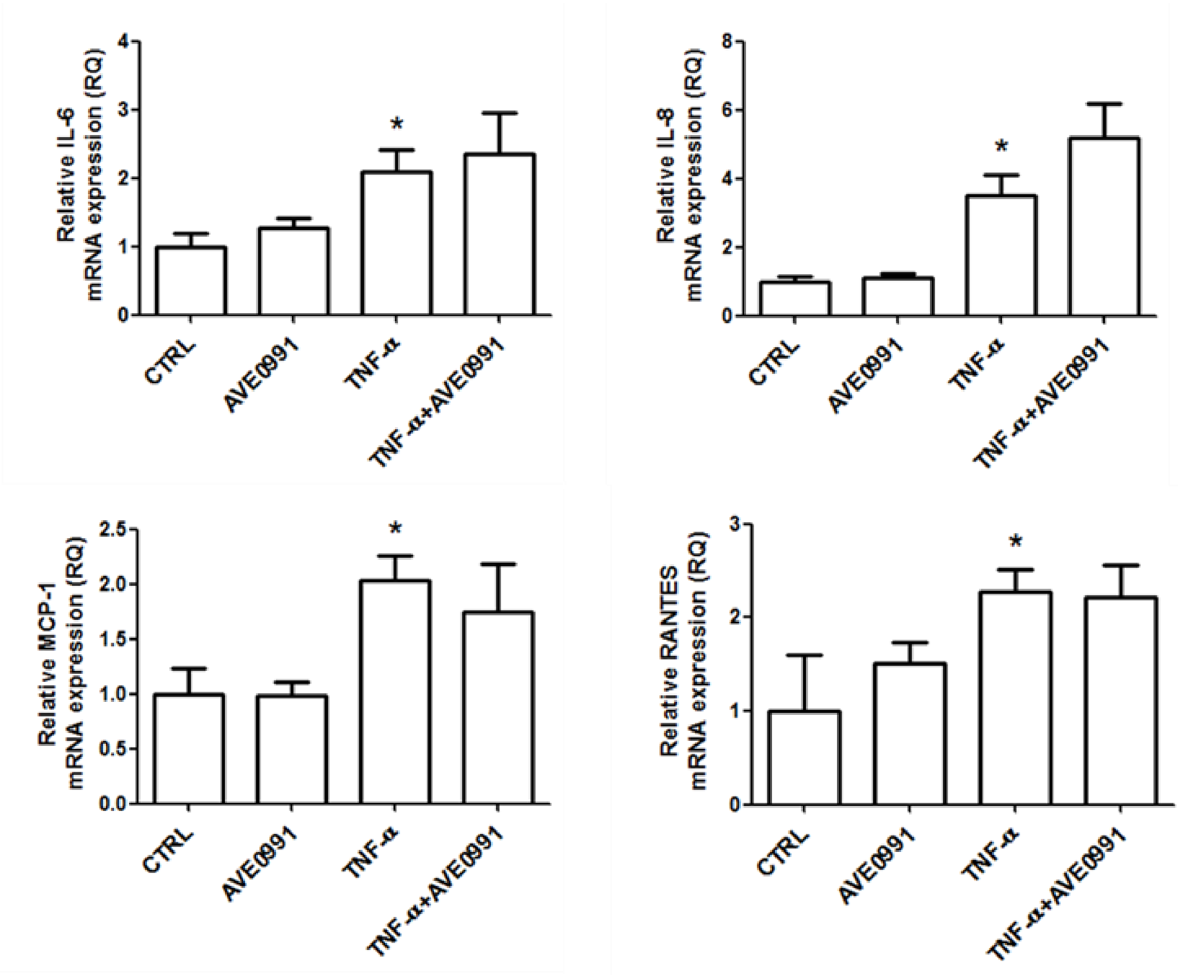

Figure Suppl. 3. Effect of AVE0991 on TNF-α activated human vascular smooth muscle cells (HVSMC) cytokine and chemokine mRNA expression. Expression of selected proinflammatory mRNA cytokines and chemokines in HVSM cells stimulated with TNF-α (10ng cm^{-3}) in presence of AVE0991 (1µM) and/or Mas receptor inhibitor A779 (5µM) (n=5). *$p<0.05$ vs CTRL (control) by T test.
